# Comparative Transcriptome Analysis Identifies Candidate Genes Related to Skin Color Differentiation in Red Tilapia

**DOI:** 10.1038/srep31347

**Published:** 2016-08-11

**Authors:** Wenbin Zhu, Lanmei Wang, Zaijie Dong, Xingting Chen, Feibiao Song, Nian Liu, Hui Yang, Jianjun Fu

**Affiliations:** 1 Freshwater Fisheries Research Centre of Chinese Academy of Fishery Sciences, Key Laboratory of Freshwater Fisheries and Germplasm Resources Utilization, Ministry of Agriculture, Wuxi 214081, China; 2 Wuxi Fisheries College, Nanjing Agricultural University, Wuxi 214081, China; 3 College of Fisheries and Life Sciences, Shanghai Ocean University, Shanghai 201306, China

## Abstract

Red tilapia is becoming more popular for aquaculture production in China in recent years. However, the pigmentation differentiation in genetic breeding is the main problem limiting its development of commercial red tilapia culture and the genetic basis of skin color variation is still unknown. In this study, we conducted Illumina sequencing of transcriptome on three color variety red tilapia. A total of 224,895,758 reads were generated, resulting in 160,762 assembled contigs that were used as reference contigs. The contigs of red tilapia transcriptome had hits in the range of 53.4% to 86.7% of the unique proteins of zebrafish, fugu, medaka, three-spined stickleback and tilapia. And 44,723 contigs containing 77,423 simple sequence repeats (SSRs) were identified, with 16,646 contigs containing more than one SSR. Three skin transcriptomes were compared pairwise and the results revealed that there were 148 common significantly differentially expressed unigenes and several key genes related to pigment synthesis, i.e. *tyr*, *tyrp1*, *silv*, *sox10*, *slc24a5*, *cbs* and *slc7a11*, were included. The results will facilitate understanding the molecular mechanisms of skin pigmentation differentiation in red tilapia and accelerate the molecular selection of the specific strain with consistent skin colors.

Coloration is an important phenotypic trait associated with multiple adaptive functions invertebrates, including thermoregulation, camouflage, social communication, selective mating and so on^1^,^2^,^3^. Coloration is determined mainly by diverse pigments synthesized by chromatophores or pigment cells. A diversity of pigment cells, associated with a series of cellular, nutritional, physiological, genetic and environmental factors, make fish skin pigmentation a complicated biological process^4^. So far, six types of pigment cells, including melanocytes (black, dark brown), xanthophores (yellow), erythrophores (red/orange), iridophores (reflecting), leucophores (white) and cyanophores (blue), have been reported in vertebrates^5^. In contrast to mammals, which possess only one type of pigment cell (themelanocyte), and amphibians and reptiles, which possess xanthophores, erythrophores and reflecting iridophores, teleost fish possess all six types of pigment cells^6^.

In mammals, the pigment melanin is the primary determinant of skin color. The molecular mechanism of melanin biosynthesis has been extensively studied and the melanogenesis pathways have been found to be conserved in vertebrates^7^,^8^. There are three types of melanin, which are eumelanin, pheomelanin and neuromelanin. Eumelanin corresponds to a brown/black color, pheomelanin corresponds to a red/yellow color, while neuromelanin is found in the brain and its function remains unknown. In the melanogenesis pathway, tyrosine is oxidized to form dopaquinone, which is then intracellularly catalyzed to become eumelanin through polymerization and oxidation reactions. However, cystine and dopaquinone can switch off the eumelanin synthesis pathway and promote the synthesis of pheomelanin^9^,^10^. The tyrosinases (*TYR*), tyrosinase-related protein 1 (*TYRP1*) and dopachrome tautomerase (*DCT*) are important enzymes in eumelanin synthesis^7^,^11^. Increased expression of microphthalmia-associated transcription factor (*MITF*) and its activation by phosphorylation stimulate the transcription of *TYR*, *TYRP1* and *DCT *7,12. In model fish, extensive studies have been conducted on zebrafish and dozens of genes have been reported to be involved in the pigmentation process through collecting and identifying the pigmentation mutations^13^,^14^,^15^. In non-model fish, transcriptome analysis in different color varieties of common carp was done to understand the genetic basis^4^,^16^. But the molecular mechanisms of fish skin color variation remain less understood. The biosynthesis of other types of pigments may involve different pathways and regulatory networks, but are in general less studied^17^.

Tilapia is the common name for nearly a hundred species of cichlid fish which originated in Africa^18^. It is currently one of the most important food fish in the world. In recent years, red tilapia is becoming more popular for aquaculture production in many parts of the world, such as China, Malaysia and Thailand^19^. While the genetic background of red tilapia varieties is not well documented, their derivation is generally attributed to the crossbreeding between mutant reddish-orange Mozambique tilapia (*Oreochromis mossambicus*) with other tilapia species like Nile tilapia (*O*. *niloticus*) and blue tilapia (*O*. *aureus*). Red tilapia has been gaining popularity due to its very fast growth, the absence of black peritoneum, salinity tolerance and adaptability to any culture system^20^. However, the pigmentation differentiation in genetic breeding and skin color variation during the overwintering period are the main problems limiting the development of commercial red tilapia culture. The pigmentation differentiation is not reversible and skin color variation during overwintering period is reversible with the environmental temperature increasing. Coloration patterns including whole pink (WP), pink with scattered black spots (PB) and pink with scattered red spots (PR) have been found in our breeding population (Fig. 1). And the market prices of PB and PR red tilapia are lower for unacceptance by consumers.

In previous study, significant genetic differences were revealed by molecular markers among the different color varieties of red tilapia, the WP grew faster than PB and PR body color types of the Malaysia red tilapia^21^. Here, we utilized the powerful approach of comparative transcriptomics analysis using next-generation sequencing and examined transcript profiles from the skins of WP, PB and PR three color Malaysia red tilapia in genetic breeding. We obtained candidate genes that may be involved in the skin pigmentation process and found a set of candidate simple sequence repeats (SSR) markers that can be used for molecular selective breeding in the future. Understanding the molecular mechanisms of skin pigmentation in red tilapia will advance our knowledge of skin color genetics in fish and accelerate the molecular selection of a specific strain with consistent skin colors.

## Results and Discussions

### Sequencing and short reads generation

To better understand fish skin color genetics, we conducted a comparative transcriptomic analysis among three skin color red tilapia, WP, PB and PR, using next-generation sequencing. Four cDNA libraries were constructed using total RNA from nine tissue samples of one red tilapia and three skin sample sets from six WP, PB and PR red tilapia individuals, respectively. Each library was sequenced on the Illumina HiSeq2500 platform. In total, high-throughput sequencing created a mean of 56,741,505 paired-end (PE) reads for each library (Table 1). After data filtering, a mean of 56,223,940 PE clean reads were obtained and the sequencing quality was high with Q20 ratio larger than 93% for all samples. All the data are available at NCBI SRA database (SRP076062).

### *De novo* assembly of the red tilapia transcriptome

All of the clean short reads were mapped to the Nile tilapia genome (*Oreochromis niloticusOrenil* 1.0) using Tophat software within known gene annotation. We found that about 82.2% of the reads could be mapped when the mismatch rate was set to 10% of a read. Based on the mapping results, the mapped reads were assembled using Cufflinks and after the correction of the differences between the assembled contigs of red tilapia and Nile tilapia transcriptome by Proovread program, a total of 67,052 contigs were obtained, corresponding to 48,870 unique genes, and their length ranged from 124 bp to 67,852 bp. In consideration of the unmapped reads which may come from the lineage-specific sequences of red tilapia transcriptome, we pooled all the unmapped reads from four libraries and *de novo* assembled them using Trinity. A total of 289,508 contigs were generated, corresponding to 213,396 unique genes with a minimum length of 200 bp, a maximum length of 10,795 bp and an N50 of 673 bp. All assemblies obtained from Cufflinks and Trinity were combined. The final assembly set, after CD-HIT removed the redundancy, consisted of 160,762 contigs, corresponding to 143,194 unique genes and their length ranged from 126 bp to 67,852 bp, with the average length of 1120.61 bp and an N50 of 2,536 bp (Table 2). There were 46,506 (28.93%) contigs longer than 1,000 bp. The detailed length distribution of contigs obtained from the Malaysia red tilapia transcriptome was in Supplementary Fig. S1.

### Functional annotation

**Figure 2:**
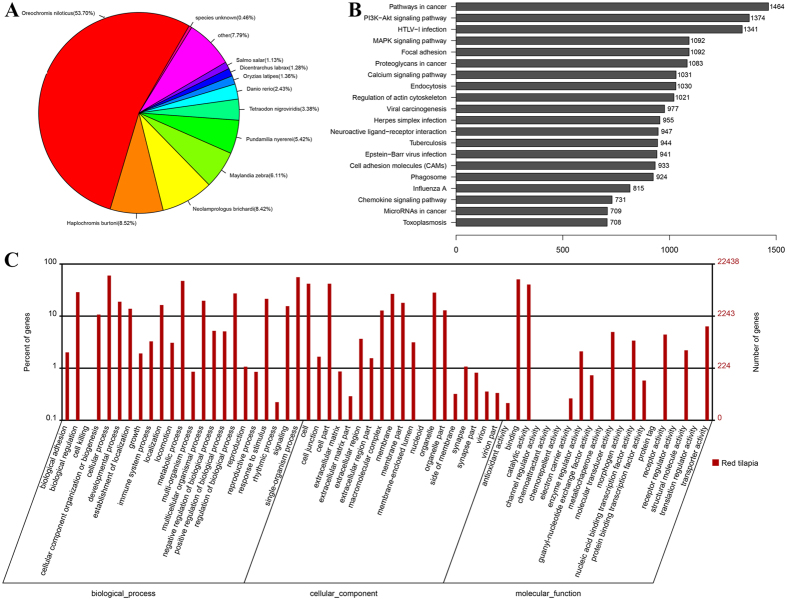
Function annotation. (**A**) Species distribution based on the best hit of blast results; (**B**) KEGG pathway classification of contigs; (**C**) Gene ontology classification of contigs.

All contigs were compared with four data bases including Nr database, the UniProt-SwissProt database, KEGG database and COG database for functional annotation, using BLASTx tools with an e-value cutoff of 1e-10. There were 88,335, 53,510, 73,763,and 13,587 assembled contigs that had significant hits against Nr, SwissProt, KEGG, and COG database, respectively (Fig. 2A–C, Table 3). Cumulatively, 88,763 assembled contigs had at least one significant hit against at least one of the four databases, corresponding to the prediction of 74,918 unique genes.

Based on the BLASTx results of the unique contigs compared with Nr database, a pie chart showed the distribution of all annotated contigs ranged from species (Fig. 2A). 91.75% contigs were annotated to 10 fish species, of which 5 species from Cichlidae and represented 82.17% of all annotated contigs. This result was consisted with the evolutionary relationship in a certain degree as the red tilapia is one of the Cichlidae fish. Among the top 10 fish species which had the homology with red tilapia, the *Oreochromis niloticus* (53.70%) had the highest number of hits, this may due to Nile tilapia was the one parent of red tilapia. The followed 4 fish species which come from Cichlidae occupied 28.47% annotated contigs. Some contigs were annotated to zebrafish (*Danio rerio*) and medaka (*Oryzias latipes*) which are model fishes and their annotations are more complete. Additionally, 0.46% of the annotated contigs had hits with unknown species, suggesting that they may represent some lineage-specific genes in red tilapia transcriptome.

**Figure 3:**
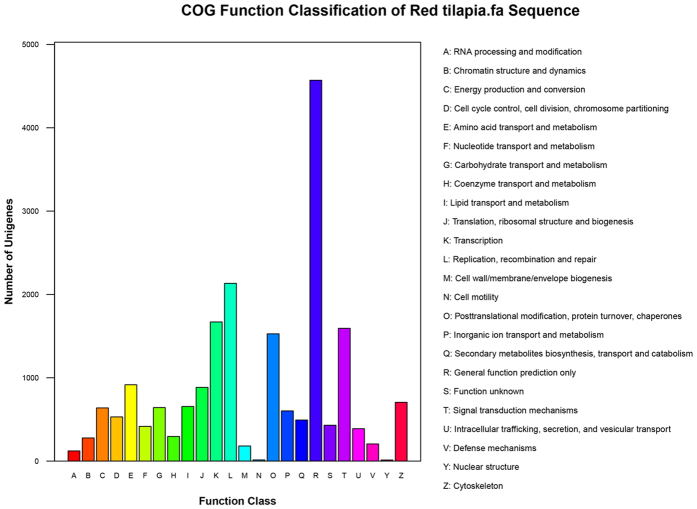


Each categorized COG term represents an ancient conserved domain, but the results implied that there is only a small proportion of contigs with annotation for COG categories. Among the functional classes, the largest cluster was the general function prediction only (4,570, 33.64%), followed by replication, recombination and repair (2,133, 15.70%), transcription (1,670, 12.29%) (see Supplementary Table S1). Only a few contigs were assigned to nuclear structure (0.10%) and cell motility (0.10%), which represented the smallest groups (Fig. 3).

To identify the biological pathway in the red tilapia transcriptome, all contigs were mapped to the KEGG database and 321 pathways were associated (see Supplementary Table S1). The top 20 pathways with the largest group of contigs are shown in Fig. 2B.

Gene ontology (GO) annotation was then performed based on the Nr annotation, 26,515 contigs (13.96%) were assigned to GO terms, corresponding to 22,439 unique genes (Table 3, Fig. 2C) (see Supplementary Table S1). As shown in Fig. 2C, a total of 62 terms were assigned, including 23 (37.10%) biological process terms, 19 (30.65%) cellular component terms and 20 (32.26%) molecular function terms. In the biological process category, cellular process (15,755) was the most abundant term, followed by single-organism process term (14,662) and metabolic process term (12,670). For the cellular component category, cell (10,906) and cell part terms (10,906) were the predominant terms and they was followed by organelle term (7,351). Within the molecular function category, binding (13,415) was the most predominant term, and it was followed by catalytic activity term (10,909) and transporter activity term (1,650).

### Assessment of transcriptome assembly

All contigs of red tilapia transcriptome were compared with Ensembl proteins of zebrafish, fugu, medaka, three-spined stickleback, tilapia and proteins of common carp download from CarpBase using BLASTx program with an e-value cutoff of 1e-5. The mapping rate of red tilapia’s contigs had significant hits on proteins ranged from 39.98% to 49.27%. And the contigs of red tilapia transcriptome had hits to 53.4% – 86.7% of the unique proteins of zebrafish, fugu, medaka, three-spined stickleback, common carp and tilapia (Table 4).

### Identification of SSRs

SSR markers are highly polymorphic and have proven to be a valuable tool for various applications in genetics and breeding. Therefore, to discover a novel set of SSRs in the contigs of red tilapia, a total of 160,762 sequences were used to determine potential microsatellite motifs by MISA. Total numbers of 77,423 SSRs were identified in 44,723 sequences, with 16,646 sequences containing more than one SSR (Table 5). On average, one SSR could be found every 4.0 kb in the red tilapia transcriptome. Taking into account that mononucleotide repeats may be a result of sequencing errors and assembly mistakes, 46,643 detected mononucleotide repeats were excluded. The most abundant type of repeat motifs was dinucleotide (24.9%), followed by trinucleotide (12.1%), tetranucleotide (2.2%), pentanucleotide (0.6%) and hexanucleotide (0.024%) repeats. Future research direction is to discover reliable markers that can be used to distinguish WP, PR and PB red tilapia. The more information of the SSRs was in the Supplementary Table S2.

### ORF identification and prediction

**Figure 4:**
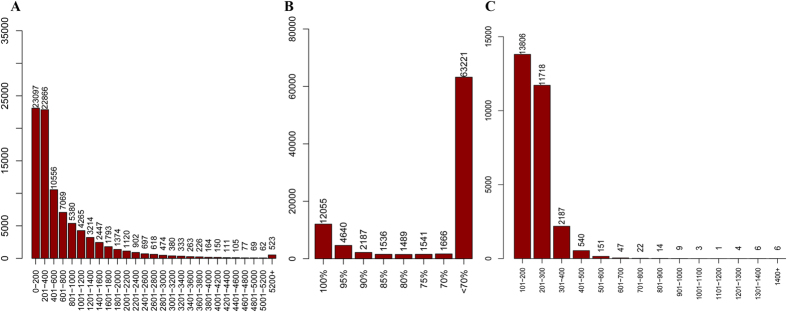
(**A**) ORF length distribution of the annotated contigs, (**B**) the integrity of the ORF region and (**C**) the predicted ORF length distribution of the unannotated contigs.

Based on the BLASTx results compared with Nr database, the Open Reading Frame (ORF) was detected from the 88,335 annotated contigs, with an average ORF length of 711.13 bp ranging from 48 bp to 35,271 bp (Fig. 4A). Among the annotated contigs, a total of 12,055 (13.64%) sequences that had 100% complete ORF region were identified (Fig. 4B), the number of the sequences that had 95% integrity ORF region was 2,187 (2.48%), and most of the sequences (63,221, 71.55%) had less than 70% integrity ORF region. To identify the potential ORF in the unannotated contigs, 72,427 contigs were analyzed by Transdecoder program. A total of 28,514 sequences were predicted containing ORF, with an average length of 220.01 bp ranging from 147 bp to 3,318 bp (Fig. 4C). The remaining 43,913 contigs contained no ORF, indicating that they may come from non-coding genes or uncompleted assembled untranslated regions (UTR).

### Comparative analysis of the skin transcriptome

**Figure 5:**
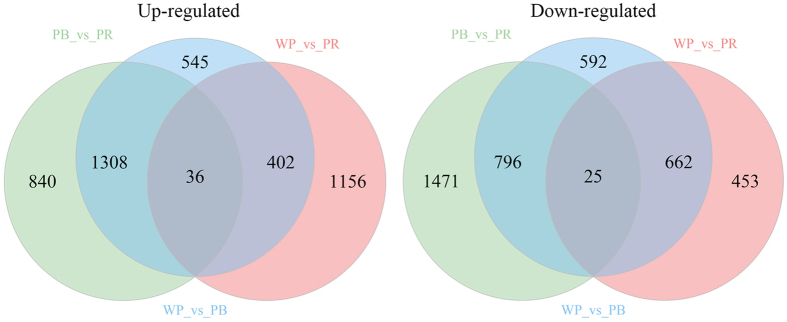
DEGs number and venn diagram of overlap of the different groups.

To reveal the differences in the skin color of red tilapia, we performed a comparative analysis of three skin transcriptomes. Based on the criteria that |logFC| ≥ 1 and FDR ≤ 0.5, we identified 4,366 differentially expressed genes in PB skin compared with WP skin, which include 2,291 up-regulated genes and 2,075 down-regulated genes in PB skin. We also identified 2,734 differential expressed genes (DEGs) in PR skin compared with WP skin including 1,594 up-regulated genes and 1,140 down-regulated genes in PR skin. In addition, 4,476 DEGs were detected in PR skin compared with PB skin, of which there were 2,184 up-regulated genes and 2,292 down-regulated genes in PR skin (Fig. 5). Further analysis indicated that a total of 148 unigenes showed significantly different expression levels in all three groups (Fig. 5). All the DEGs were listed in the Supplementary Table S3.

**Figure 6:**
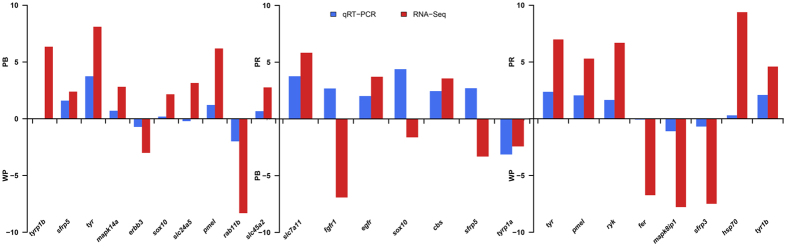
Comparison of gene expression patterns obtained using RNA-Seq and qRT-PCR. Log-fold changes are expressed as the ratio of gene expression after normalization to β-actin.

To validate the differentially expressed genes identified by comparative transcriptomic analysis, we selected 20 genes which were pigment biosynthesis related genes for qRT-PCR confirmation of differential expression from three comparative groups. As shown in Fig. 6, the qRT-PCR expression patterns of 16 out of 20 randomly selected DEGs were in agreement with the results of RNA-Seq analysis. In addition, we chose 9 out of the 20 genes and gene β-actin for validation of RNA-Seq assembly accuracy, the Sanger sequencing results showed that PCR products were consistent with the assembly sequences.

All these genes are involved in the production of melanin, which is the substance that gives skin, hair, and eyes their color. The most two common types of melanin are eumelanin and pheomelanin, which correspond to brown/black color and red/yellow color respectively. In the comparative transcriptome analysis of PB skin and WP skin, it was obvious that the amount and density of eumelanin were much higher in PB skin than in WP skin, and the results of DEGs analysis corresponded to observed result. The expression level of tyrosinase-related protein 1 (*tyrp1*), sex determining region Y-box 10 (*sox10*), premelanosome protein (*pmel*/*silv*), solute carrier family 24 (sodium/potassium/calcium exchanger) member 5 (*slc24a5*), solute carrier family 45 member 2 (*slc45a2*) and tyrosinase (*tyr*) were up-regulated in PB skin, implying that these genes play a key role in the contribution to black coloration in red tilapia in the eumelanin synthesis pathway. *tyrp1*, *pmel* and *tyr* are involved in the production of melanin, their characters and functions were reported a lot in other vertebrate species and teleost fishes^11^,^22^,^23^. A gene we should take a note of is *sox10*, which belongs to a family of genes that play a critical role in the formation of tissues and organs during embryonic development. *sox10* can activate the *mitf* and raise *mitf* expression^24^ Increased expression of *mitf* and its activation stimulate the transcription of *tyr*, *tyrp1* and *dct*^7^,^12^, which are responsible for the synthesis of melanin. Another significantly differentially expressed gene was *slc24a5*. This protein has a major influence on natural skin color variation in humans, the removal of which disrupts melanogenesis in human and mice melanocytes, causing a significant reduction in melanin pigment production^25^. Consistent with these studies, our results showed that the expression level of *slc24a5* was 8.88-fold up-regulated in PB skin when compared with WP skin. While investigating the DEGs list for the comparison between PR skin and WP skin, we also found that the expression level of *tyrp1*, *pmel* and *tyr* genes were up-regulated in PR skin, the results again verified that these genes are important in the production of melanin again.

Lastly, we compared the PR skin and PB skin transcriptome and found that *tyrp1* and *sox10* genes were up-regulated in the PB skin. Another gene that should be taken note of is *cbs* (cystathionine beta synthase), also known as cysteine synthase. The protein encoded by this gene performs a crucial role in the biosynthetic pathway of cysteine. The *cbs* gene was up-regulated in the PR skin, suggesting that more cysteine was synthesized, which was in agreement with our results that high-level expression of *cbs* contributed to the production of pheomelanin^26^. It is well established, cysteine is involved in the biosynthetic pathway for pheomelanin. In the presence of cysteine, a sulphur-rich amino-acid, DOPA-quinone is transformed into cysteinyl-DOPA, an intermediary in the synthesis of pheomelanin^26^. Some studies suggested that *slc7a11*/*xCT*, a cysteine/glutamate exchanger, can directly affects the pheomelanin synthesis^4^,^27^,^28^. In our results, we found that the gene *slc7a11* was assembled into several assemblies, the differential expression analysis showed that there was no significant expression difference. But when we use the annotation genes of tilapia to identify the DEGs directly, the results showed that the *slc7a11* was significantly up-regulated in PR skin. It is therefore again confirmed that the *slc7a11* plays a key role in the production of pheomelanin.

**Figure 7:**
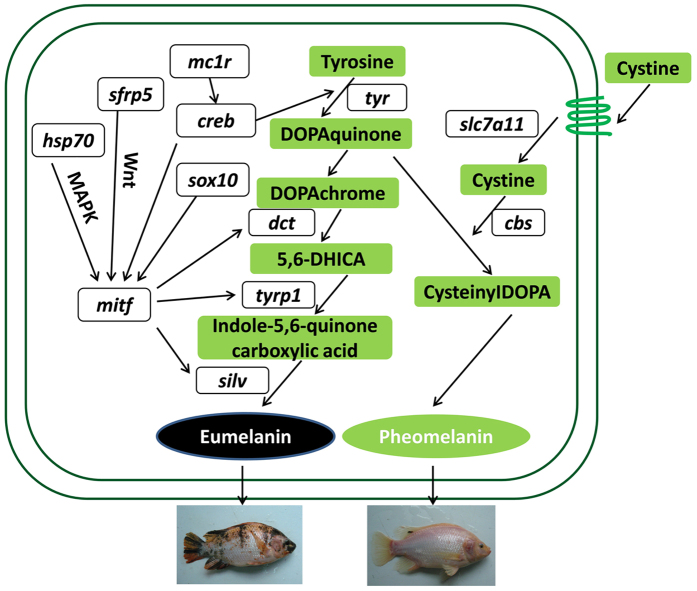
The putative genes and pathways involved in the red tilapia skin pigmentation process.

Melanogenesis is under complex regulatory control by multiple agents interacting via pathways. It is activated by receptor-dependent mechanisms in hormonal, auto-, para-, or intracrine fashion^29^. The most important positive regulator of melanogenesis is the MC1 receptor with its ligands melanocortic peptides determining intensity of melanogenesis and the type of synthesized melanin^29^. *mc1r* activates the cyclic AMP (cAMP) response-element binding protein (*creb*), and its cascade involves the up-regulation of the expression of microphthalmia associated transcription factor (*mitf*), which could bind and activate melanogenic gene promoters and increase their expression, resulting in increased melanin synthesis^30^. The putative genes and pathways involved in the red tilapia skin pigmentation process are showed in Fig. 7. Briefly, *mc1r* positive regulates cAMP expression levels resulting in melanin synthesis process. The synthesis of eumelanin is then mediated by *tyr*, *sox10*, *pmel*, *slc24a5* and *slc45a2*. The pheomelano genesis during melanin biosynthesis may depend on the presence of *cbs*, which synthesizes the cysteine and consequently promote the synthesis of pheomelanin.

### Enrichment and pathway analysis of DEGs

In the further analysis of the GO term enrichment and KEGG pathway of DEGs, all the DEGs were classified into different gene ontologies and pathway (see Supplementary Table S4). The results showed that many DEGs were assigned to pigmentation-related term, such as melanin biosynthetic process (GO:0042438), melanin metabolic process (GO:0006582) and melanosome organization (GO:0032438). The genes enriched in these pigmentation-related terms were informative and worth further study.

The KEGG pathway analysis results showed that some DEGs were associated with pigmentation-related pathways. We focused on the melanogenesis, Wnt signaling pathway and MAPK signaling pathway. It’s reported that, in vertebrates both Wnt and MAPK signaling pathways were involved in pigment cell development^31^. In our study, some identified DEGs were enriched in the Wnt or MAPK signaling pathway, and they are likely to be involved in melanin synthesis in red tilapia (see Supplementary Table S4). For example, *sfrp5* (secreted frizzled-related protein 5), acts as a soluble modulator of Wnt signaling pathway (Fig. 7). It’s reported that *sfrp5* is highly expressed in the retinal pigment epithelium^32^, corresponding to the findings of this study that the expression of *sfrp5* is up-regulated in PR and PB skin. Another gene *hsp70*, member of the heat shock proteins, is involved in the MAPK signaling pathway (Fig. 7). Recent studies suggest that the roles of the *hsp70* molecular chaperone and proteasomal and lysosomal proteolytic pathways are evaluated in human retinal pigment epithelium^33^. In our results, *hsp70* was expressed higher in PR skin compared to WP skin, suggesting that *hsp70* may be involved in the skin pigmentation process. The details of interactions of the melanogenetic pathway with other regulatory pathways such as nervous system, immune system or circulatory system in red tilapia skin remain to be further investigated.

## Materials and Methods

### Ethics statement

This study was approved by the Animal Care and Use committee of the Centre for Applied Aquatic Genomics at the Chinese Academy of Fishery Sciences. The methods of all experiments were carried out in accordance with the Guide for the Care and Use of Experimental Animals of China.

### Sample collection

Red tilapia were obtained from the Qiting Pilot Research Station (Yixing, Jiangsu, China), which are affiliated to the Freshwater Fisheries Research Center (FFRC), Chinese Academy of Fishery Sciences. Fish (initial weight: 53.1 ± 1.30 g) was maintained in conical fibreglass tanks (water depth: 50 cm, volume: 256 L) in a flow-through water system during the acclimation and experimental period. The water temperature was maintained at 27 ± 1 °C, pH = 7–8, Dissolved oxygen (DO) > 6 mg L^−1^ and NH_4_-N < 0.5 mg L^−1^. Aeration was supplied to each tank 24 h per day and photoperiod was 12D:12L.

Nine tissue samples including brain, muscle, liver, intestine, heart, kidney, ovary, skin, and spleen were collected from one red tilapia. Another three sets of skin tissues were collected from six WP red tilapia, six PB (pink with 2/3 or above 2/3 scattered black spots of all skin) red tilapia and six PR (pink with 2/3 or above 2/3 scattered red spots of all skin) red tilapia individuals, respectively. And the skins were sampled from the back skin with black spot and red spot regions. All fresh tissue samples were frozen immediately in liquid nitrogen and then stored at −80 °C before RNA isolation.

### RNA isolation, cDNA library construction and sequencing

Total RNA was obtained from red tilapia samples using TRIzol (Invitrogen, UK) according to the manufacturer’s protocol. Then genomic DNA was removed from RNA sample using DNase (New England Biolabs). RNA purity was assessed using the Nanodrop-2000 (Thermo Scientific, USA). Each RNA sample had an A260:A280 ratio above 1.9 and A260:A230 ratio above 1.8. Total RNA integrity was then subsequently checked using an Agilent Technologies 2100 Bioanalyzer with an RNA Integrity Number (RIN) value greater than or equal to 8. An equal amount of total RNA from nine tissue samples was pooled, and an equal amount of total RNA from six individuals of each color group (WP, PB and PR red tilapia) was pooled separately. Next, four sequencing libraries were constructed by TruSeq™RNA Sample Preparation Kit according to the product instruction (Illumina). Each library was sequenced using Illumina HiSeq2500 for 2 × 125 bp pair-end (PE) sequencing.

### *De novo* assembly of reference sequences

Quality control of all raw reads was conducted by Fastqc (http://www.bioinformatics.babraham.ac.uk/projects/fastqc/) software. An initial filtering step was performed to exclude poor quality reads, including adaptor reads, ambiguous nucleotides and low-quality reads (reads having more than 50% bases with quality value). The clean reads were first mapped onto the Nile tilapia reference genome^34^ (*Oreochromis_niloticus*.*Orenil*1.0) by Tophat^35^ (v2.0.10). The ‘−G’ option of Tophat together with the Gene Transfer Format (GTF) file of the Ensemble gene annotation was used for read mapping. Considering that the red tilapia is not exactly the same as the Nile tilapia, the mismatch rate of a read was set to 10% (–*segment-mismatches 3 -N 12*). The mapped reads were then assembled using Cufflinks^36^ (V2.2.1) with default parameters. To get high-confidence isoforms, only those assembled transcripts with FPKM > 0 were retained for further analyses. Then we extracted the assembled transcripts sequence from the tilapia genome sequence according the GTF file generated by Cufflinks. Considering that the transcriptome of red tilapia was not exactly same with Nile tilapia, we corrected the difference of those assembled transcripts with high quality Illumina short reads. Proovread^37^ a software which was designed to correct erroneous long reads sequenced by SMRT (Single Molecule Real-Time) with high quality short reads data as generated by Illumina sequencers, was used to correct the differences between the contigs of red tilapia and the transcriptome of tilapia. The most accurate correction results were used to perform the downstream analysis. Thirdly, we extracted the unmapped reads from the unmapped BAM file, and *de novo* assembly of the unmapped reads was performed using Trinity^38^ (version trinityrnaseq-2.0.2) with default parameters.

The corrected assembled sequences generated by Cufflinks and sequences output from Trinity software were then combined. Cd-hit^39^ was used to reduce redundancy of the combined assembled sequences. And the resulting contigs were considered as the red tilapia reference sequences.

### Assembled sequence annotation and classification

Functional annotation of the assembled reference sequences was performed by homology searches against the NCBI Nr(Non-redundant protein)^40^ database, the UniProt-SwissProt (The Universal Protein Resource)^41^ database, the COG (Cluster of Orthologous Groups of protein)^42^ database and the KEGG(Kyoto Encyclopedia of Genes and Genomes)^43^ database. Searches were conducted by the BLASTx^44^ program with an e-value cutoff of 1e-10. The gene name and description of the best blast hit was assigned to each contig with significant hits.

### Assembly assessment

To compare the similarity to other teleost species, all assembled sequences were compared to Ensembl proteins of zebrafish (*Danio rerio*), fugu (*Takifugu rubripes*), medaka (*Oryzias latipes*), three-spined stickleback (*Gasterosteus aculeatus*) and Nile tilapia (*Oreochromis niloticus*) and proteins of the gynogenetic Songpu common carp (*Cyprinus carpio*) download from CarpBase^27^, by using BLASTx program with an e-value cutoff of 1e-5.

### SSRs identification

All contigs were used to identify SSR markers by a microsatellite identification program, MIcroSAtellite (MISA) (http://pgrc.ipk-gatersleben.de/misa/misa.html). The parameters used to identify simple sequence repeats were at least for 6 repeats for di-nucleotide and 5 repeats for tri-, tetra-, penta- and hexa-nucleotide. The compound repeats which composed of two or more microsatellite sequences separated by 100 bases were identified.

### ORF identification and prediction

The ORF regions of the contigs which were annotated with Nr database were identified by in-house Perl scripts. Putative ORFs of the unannotated contigs were predicted by Transdecoder (http://transdecoder.sourceforge.net/) program. We defined a contig which has full-length ORF region while it can cover the entire length of the subject protein. Otherwise we calculated the subject protein coverage as the completeness percentage.

### Differential gene expression analysis

Clean reads of the skin tissues of each color were aligned to the assembled reference by Bowtie^45^, and then RSEM^46^ program was used to estimate and quantify gene and isoform abundances. Gene expression was measured in fragments per kilobase of exon per million reads mapped (FPKM). Finally, edger^22^ was used to normalize the expression level of each gene in 3 skin samples to identify the differentially expressed genes by pairwise comparisons. The threshold values of |logFC|≥1 and FDR (False Discovery Rate) ≤0.05 were used to judge the significance of DEGs. GO term enrichment and KEGG pathway analysis of DEGs was performed using KOBAS program.

### qRT-PCR analysis

qRT-PCR was performed on the ABI PRISM 7500 Real-time PCR System. The amplifications were performed in a total volume of 10 μl and included 5 μl of 2X SYBR Green MasterMix reagent, 1 μl of cDNA and 0.2 μl of each primer (10 μM). The thermal cycling profile consisted of an initial denaturation at 95 °C for 5 min followed by 40 cycles of denaturation at 95 °C for 15 s and annealing/extension at 60 °C for 45 s. An additional temperature-ramping step from 95 °C to 65 °C was used to produce the melting curve. All reactions were conducted in triplicate and included negative controls with no template. Values were determined based on two biology replicates, each with three technical replicates. The expression levels of genes were normalized to the levels of β-actinin the same sample. Two-side *t* test was used to compare expression levels.

## Additional Information

**How to cite this article**: Zhu, W. *et al*. Comparative Transcriptome Analysis Identifies Candidate Genes Related to Skin Color Differentiation in Red Tilapia. *Sci. Rep.* **6**, 31347; doi: 10.1038/srep31347 (2016).

## Figures and Tables

**Figure 1:**
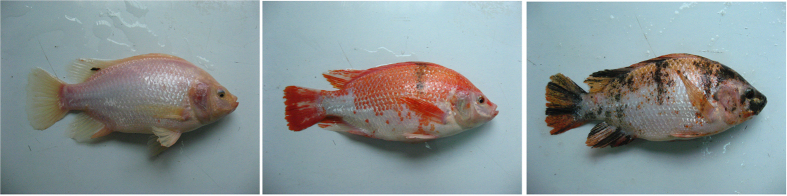
Three skin color types of Malaysia red tilapia. (WP: whole pink, PR: pink with scattered red spots and PB: pink with scattered black spots from left to right respectively).

**Table 1:** Summary of the sequencing data from Malaysia red tilapia.

Sample	Number of raw reads	Number of clean reads
TM*	97,254,220	96,114,122
WP*	43,209,760	42,899,842
PB*	44,848,770	44,537,864
PR*	41,653,270	41,343,930
Total reads	226,966,020	224,895,758

^*^Skin color varieties of red tilapia: WP-whole pink, PB-pink with scattered black spots, PR-pink with scattered red spots. TM-tissues mix sample.

**Table 2:** Overall assembly statistics for the transcriptome of Malaysia red tilapia.

Item	Genome-guided assembly	*De novo* assembly of unmapped reads	Final reference assembly
Total number of contigs	67,052	289,508	160,762
Total number of genes	48,870	213,396	143,194
Total length of contigs (bp)	156,098,806	156,998,080	180,151,466
Maximum contig length	67,852	10,795	67,852
Minimum contig length	124	200	126
Average contig length	2328.03	542.29	1120.61
N25	5,852	1,297	4,979
N50	3,760	673	2,536

**Table 3:** Statistics of function annotation.

Annotated database	Annotated number	%
Nr	88,335	54.95%
Swiss-Prot	53,510	33.29%
COG	13,587	8.45%
GO	26,515	16.49%
KEGG	73,763	45.88%
All annotated contigs	88,763	55.21%
All contigs	160,762	100.00%

**Table 4:** Summary of BLASTx search results of Malaysia red tilapia transcriptome.

Database	Red tilapia hits	% of total red tilapia contigs	Unique protein	% of total unique proteins
Zebrafish	67,665	42.09%	23,739	53.4% of 44,487
Fugu	64,269	39.98%	26,103	54.6% of 47,841
Medaka	69,391	43.16%	19,321	78.3% of 24,674
Three-spine stickleback	66,905	41.62%	21,046	76.3% of 27,576
Common carp	68,069	42.34%	25,716	55.2% of 46,609
Tilapia	79,203	49.27%	23,192	86.7% of 26,763

**Table 5:** Distribution of SSRs based on the number of repeat units.

Repeat numbers	SSR type						Total	Percentage
	Mono-	Di-	Tri-	Tetra-	Penta-	Hexa-		
5	0	0	5,013	845	250	12	6,120	7.90%
6	0	5,555	2,045	435	64	5	8,104	10.47%
7	0	3,022	1,009	106	34	0	4,171	5.39%
8	0	1,855	583	76	29	0	2,543	3.28%
9	0	1,372	205	58	25	2	1,662	2.15%
10	13,251	1,081	121	39	19	0	14,511	18.74%
11	8,163	1,040	90	35	13	0	9,341	12.06%
12	5,523	924	61	17	2	0	6,527	8.43%
>12	19,706	4,415	215	96	12	0	24,444	31.57%
Total	46,643	19,264	9,342	1,707	448	19	77,423	100.00%
	60.24%	24.88%	12.07%	2.20%	0.58%	0.02%	100.00%	
