# Erratum: Comparative Transcriptome Analysis Identifies Candidate Genes Related to Skin Color Differentiation in Red Tilapia

**DOI:** 10.1038/srep46979

**Published:** 2018-05-11

**Authors:** Wenbin Zhu, Lanmei Wang, Zaijie Dong, Xingting Chen, Feibiao Song, Nian Liu, Hui Yang, Jianjun Fu

Scientific Reports 6: Article number: 3134710.1038/srep31347; published online: 08 11 2016; updated: 05 11 2018

In the original version of this Article, the Supplementary Information files containing the supplementary figure and datasets were omitted. This error has now been corrected in the PDF and HTML versions of the Article.

